# Apoptosis-Inducing Antitumor Activity of Curcumin-Loaded PCL Microfibers Incorporating Nano-Hydroxyapatite for Breast Cancer Therapy

**DOI:** 10.1021/acsomega.5c10882

**Published:** 2026-04-27

**Authors:** Y. Emre Bulbul, Hayrani Eren Bostancı, Yıldırımcan Demirtaş, Hüsnü Çağrı Genç, Muzeyyen Asli Ergozoglu, Funda Demirtas-Korkmaz, Ş. Melda Eskitoros-Togay

**Affiliations:** † Department of Chemistry, Faculty of Engineering and Natural Sciences, Suleyman Demirel University, Isparta 32220, Turkey; ‡ Department of Biochemistry, Faculty of Pharmacy, Sivas Cumhuriyet University, Sivas 58140, Turkey; § Department of Surgical Medical Sciences, Division of General Surgery, Faculty of Medicine, Sivas Cumhuriyet University, Sivas 58140, Turkey; ∥ Department of Medical Oncology, Faculty of Medicine, Çukurova University, Adana 01790, Turkey; ⊥ Department of Medical Biology, Faculty of Medicine, Giresun University, Giresun 28100, Turkey; # Department of Pharmacy Services, Gulhane Vocational School of Health Services, University of Health Sciences, Ankara 06010, Turkey

## Abstract

Breast cancer remains one of the most prevalent and life-threatening cancers worldwide, emphasizing the need for innovative therapeutic strategies that enable localized and sustained drug delivery. In this study, hybrid electrospun fibrous mats composed of poly­(ε-caprolactone) (PCL), nanohydroxyapatite (n-HAp), and curcumin (Cur) were successfully fabricated and evaluated for breast cancer treatment. The mats were produced by electrospinning with n-HAp concentrations of 1%, 3%, and 5% (w/w), and a fixed Cur content of 5% (w/w). Morphological analysis revealed uniform, randomly oriented, and bead-free fibers with average diameters of 2.34 ± 0.31 μm, 1.30 ± 0.90 μm, 1.10 ± 0.40 μm, and 1.50 ± 0.90 μm for PCL/Cur, PCL/1HAp-Cur, PCL/3HAp-Cur, and PCL/5HAp-Cur, respectively. FTIR and XRD analyses confirmed the successful incorporation of Cur and n-HAp into the PCL matrix without chemical degradation, while partial amorphization of the additives enhanced their dispersion within the fibers. Contact angle results indicated increased hydrophilicity with higher n-HAp content, reaching the lowest value (112 ± 1.8°) for PCL/3HAp-Cur. In vitro drug release experiments demonstrated a biphasic release behavior, with cumulative Cur release after 24 h of approximately 20%, 30%, 60%, and 80% for PCL/Cur, PCL/1HAp-Cur, PCL/3HAp-Cur, and PCL/5HAp-Cur, respectively. The release kinetics were best fitted to the Weibull model, indicating a diffusion-controlled mechanism. Biological evaluations revealed that PCL/3HAp-Cur exhibited the strongest anticancer activity, reducing MDA-MB-231 breast cancer cell viability to ∼45% after 48 h, while maintaining high biocompatibility with L929 fibroblast cells (>87% viability). Annexin V/PI flow cytometry confirmed apoptosis as the dominant mode of cell death. Overall, the findings demonstrate that PCL/3HAp-Cur nanofibrous mats offer an optimal balance of structural uniformity, sustained Cur release, enhanced hydrophilicity, and selective anticancer efficacy, making them promising candidates for localized breast cancer therapy.

## Graphical abstract

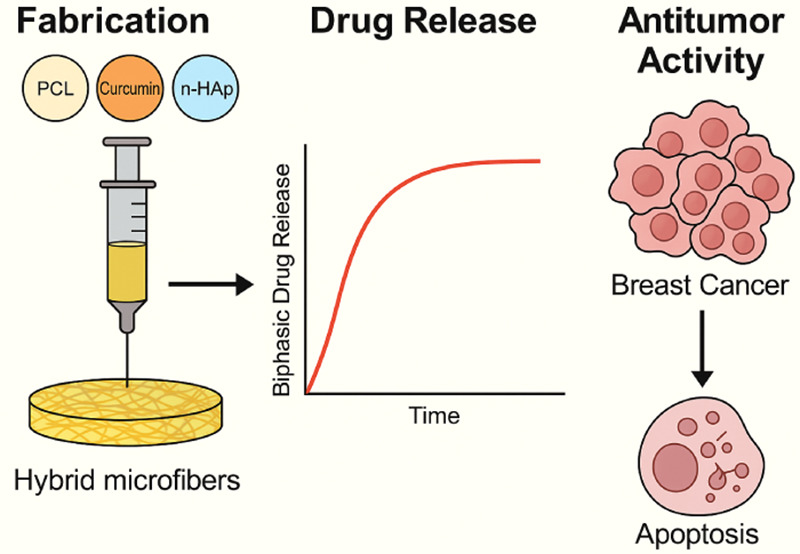

## 1 Introduction

Breast cancer remains one of the most prevalent malignancies worldwide and continues to pose a major global health challenge, primarily due to its high recurrence rate and resistance to conventional therapies. Standard treatment modalities, including surgery, chemotherapy, and radiotherapy, often suffer from systemic toxicity, poor selectivity, and limited therapeutic efficiency.^1–3^ These limitations have driven increasing interest in developing innovative drug delivery systems (DDS) capable of providing localized, controlled, and sustained release of anticancer agents while minimizing off-target effects.^4,5^

Biodegradable and biocompatible polymers have emerged as promising materials for constructing DDS platforms. Among them, poly­(ε-caprolactone) (PCL) has gained considerable attention because of its excellent mechanical strength, slow degradation rate, and high drug compatibility.^6,7^ The incorporation of curcumin, a natural polyphenolic compound derived from *Curcuma longa*, further enhances the therapeutic potential of polymeric DDS. Curcumin exhibits well-documented antitumoral, antiinflammatory, antioxidant, and pro-apoptotic activities against various cancer types, including breast cancer. However, its clinical application is limited by poor aqueous solubility, rapid metabolism, and low bioavailability.^8,9^ Embedding curcumin into a PCL matrix via electrospinning offers a means to overcome these drawbacks by improving its stability and enabling a sustained and site-specific release profile.^10^

In this context, recent advancements in anticancer treatments emphasize targeted and localized drug delivery systems that enhance therapeutic efficacy while minimizing systemic toxicity. Nanomedicine approaches, particularly those employing PCL composites combined with curcumin, have shown potential for improved tumor-specific accumulation and prolonged release profiles, thereby increasing curcumin’s bioavailability and antitumoral effects against various cancers.^11–13^ Specifically, PCL-based scaffolds facilitate sustained release and localized therapy, significantly reducing adverse effects compared to conventional chemotherapy techniques.^14,15^ The integration of hybrid systems, such as the PCL-curcumin formulations reported in various studies, highlights their potential in enhancing apoptotic activity in cancer cells and supporting innovative therapeutic strategies for effective cancer management.^16–18^

To further enhance therapeutic efficiency, the incorporation of nanohydroxyapatite (n-HAp) nanoparticles into polymer matrices has attracted growing attention.^19,20^ n-HAp, known for its bioactivity, biocompatibility, and osteoconductivity, provides an ideal environment for cell adhesion and proliferation.^21^ Importantly, recent evidence indicates that n-HAp can also exert intrinsic anticancer effects and interact favorably with both hydrophilic and hydrophobic drug molecules, thereby improving encapsulation efficiency and regulating release kinetics.^22^ These features make n-HAp an excellent additive for constructing a multifunctional DDS for cancer therapy.

In our previous work, PCL/PEO composite nanofibers containing low concentrations of n-HAp (0.1–0.5 wt %) and curcumin were successfully fabricated by electrospinning. The incorporation of 0.3 wt % n-HAp provided the most stable release behavior and exhibited the highest cytotoxicity against MCF-7 breast cancer cells, demonstrating that the interaction between n-HAp content and drug release behavior was nonlinear.^23^ However, further optimization was required to elucidate the influence of higher n-HAp concentrations and evaluate apoptosis induction associated with the anticancer effects.

Building upon these findings, this study introduces several distinctive aspects. First, electrospun hybrid fibrous mats containing higher n-HAp concentrations (1, 3, and 5 wt %) were prepared and systematically compared. Second, flow cytometry was employed to characterize apoptosis induction and cell cycle alterations, offering deeper mechanistic insights into anticancer activity. In addition, water contact angle measurements were conducted to evaluate surface wettability and its potential effect on cell–material interactions. Finally, the release kinetics of curcumin were analyzed using multiple mathematical models to better describe the diffusion mechanisms governing sustained release.

By integrating these design elements, this research aims to provide a comprehensive understanding of how the n-HAp content modulates the structural, physicochemical, and biological properties of hybrid PCL/Cur nanofibers. The outcomes are expected to guide the optimization of electrospun DDS platforms for effective, targeted, and biocompatible breast cancer therapy, while contributing to the broader field of polymer-based nanobiomaterials.

## 2 Materials and Methods

### 2.1 Materials

Poly­(ε-caprolactone) (PCL, average molecular weight 80,000), nanohydroxyapatite (n-HAp, particle size <200 nm, BET), curcumin (≥95% total curcuminoid content, extracted from *Curcuma longa* rhizome), phosphate-buffered saline (PBS) tablets, Tween-80, chloroform (≥99% purity), *N,N*-dimethylformamide (DMF), fetal bovine serum (FBS), Dulbecco’s modified Eagle’s medium (DMEM), penicillin–streptomycin solution, l-glutamine, and thiazolyl blue tetrazolium bromide (MTT; 3-(4,5-dimethylthiazol-2-yl)-2,5-diphenyltetrazolium bromide) were all purchased from Sigma-Aldrich (St. Louis, USA). All chemicals were of analytical grade and were used as received without further purification.

### 2.2 Fabrication of Fibrous Material Systems by Electrospinning

Electrospinning solutions were prepared by dissolving 18% (w/v) PCL in a DMF/chloroform (1:9, v/v) solvent system under magnetic stirring for 6 h at room temperature until a clear, homogeneous solution was obtained. Different concentrations of n-HAp (1%, 3%, and 5%, w/v) were dispersed in the polymer solution by ultrasonication for 30 min to ensure a uniform n-HAp dispersion within the polymer solution. Subsequently, curcumin (5%, w/w, relative to polymer weight) was incorporated into each mixture under continuous stirring for 2 h in the dark to avoid photodegradation. The compositions of the solutions were decided in our previous study.^10^ Table 1 presents the compositions of the prepared solutions for the electrospun mats.

**Table 1:** Composition of PCL-Based Electrospinning Solutions

Code	Solutions	nHAp % (w/v)	Curcumin % (w/w)
a	PCL/Cur	0	5
b	PCL/1 nHAp-Cur	1	5
c	PCL/3 nHAp-Cur	3	5
d	PCL/5 nHAp-Cur	5	5

For the electrospinning process, the prepared solutions were electrospun using a 5 mL syringe fitted with a 21-gauge metallic needle. The process parameters were optimized as follows: a voltage of 20 kV, a flow rate of 1.2 mL/h, and a needle-to-collector distance of 15 cm. The fibers were collected as nonwoven mats on grounded aluminum foil. All electrospinning processes were performed at room temperature and under ambient humidity. Representative macroscopic photographs of the electrospun hybrid microfiber mats are presented in Scheme 1, confirming homogeneous fabrication prior to detailed characterization.

**Scheme 1:**
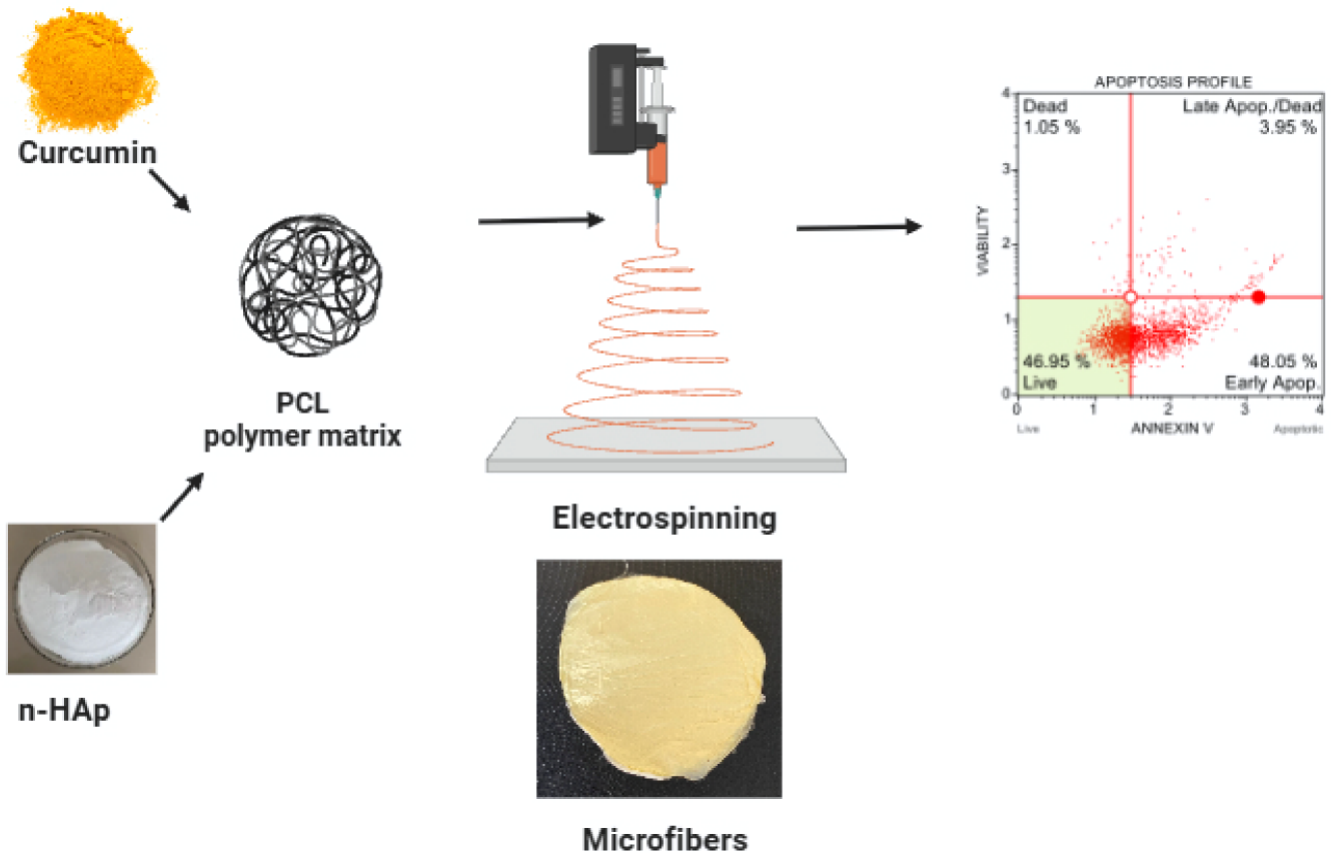
Schematic Illustration of Curcumin-Loaded PCL/n-HAp Hybrid Microfiber Fabrication via Electrospinning and the Proposed Apoptosis-Mediated Anticancer Mechanism

### 2.3 Morphological and Physicochemical Characterizations

For analyzing the fiber diameter and the morphology of the electrospun mats, a scanning electron microscope (SEM, JSM 6060 LV) by JEOL Ltd. (Tokyo, Japan), operated at an accelerating voltage of 5–10 kV, was utilized. All the samples were separately placed onto an SEM grid and coated with gold under vacuum conditions by using a Polaron SC 502 sputter coater before the observations. Digimizer Image Analysis Software (MedCalc Software; BVBA, Ostend, Belgium) was used to measure the average diameters of electrospun mats from 50 fibers from the SEM image.

The chemical bonding and phase state of the electrospun mats were characterized by Fourier-transform infrared spectroscopy (FT-IR, Nicolet Avatar 370; Thermo Fisher Scientific, Inc., Waltham, MA, USA) in the range of 4000–500 cm^–1^, and X-ray diffraction (XRD, APD 2000 Pro diffractometer, GNR, USA) at a 2θ range of 5–40°, 40 kV, and 30 mA with Cu Kα radiation, respectively. The surface wettability of the electrospun mats was assessed by measuring the static water contact angle (θ) using a goniometer (Attension Theta Lite, Biolin Scientific, Finland). Distilled water droplets (5 μL) were gently placed on the mat surfaces at room temperature, and the contact angle was recorded within 10 s using image analysis software. Each measurement was repeated three times at different positions, and the results were expressed as the mean ± standard deviation (SD). The test aimed to evaluate the influence of the n-HAp content on the surface hydrophilicity of the fibers.

### 2.4 Encapsulation Efficiency and Drug Release Study

To calculate the encapsulation efficiency (%) of curcumin in the mats, the UV–vis Spectrophotometer (Shimadzu UV-1601, Kyoto, Japan) method was used to obtain a predetermined calibration curve. The encapsulation efficiency was calculated as the ratio between the actual loading content and the theoretical content of curcumin by the following eq 1.^24^

1 $$\mathrm{Encapsulation}\;\mathrm{efficient}\left(\%\right)=\frac{\mathrm{Actual}\;\mathrm{content}\;\mathrm{of}\;\mathrm{curcumin}}{\mathrm{Theoretical}\;\mathrm{content}\;\mathrm{of}\;\mathrm{curcumin}}\times 100$$

*In vitro* release study was carried out according to our previous methods^10^ by using a UV–vis spectrophotometer. To conduct this experiment, 1 cm × 1 cm samples were submerged in 30 mL of phosphate buffer saline (PBS) solution with a pH of 7.4 at 37 °C, which included 0.5 wt % of Tween 80. At predetermined time intervals, a 1 mL solution was taken to obtain the absorbance value using a UV spectrophotometer at 420 nm. The fresh solution was put back into the main solution. The procedure was performed in triplicate, and the standard deviations were expressed. The cumulative release of curcumin was calculated by the following eq 2:

2 $$\mathrm{Cumulative}\;\mathrm{release}\;(\%)=\frac{\mathrm{Amount}\;\mathrm{of}\;\mathrm{curcumin}\;\mathrm{release}}{\mathrm{Amount}\;\mathrm{of}\;\mathrm{initial}\;\mathrm{curcumin}}\times 100$$

### 2.5 Anticancer Activity

#### 2.5.1 Cell Culture

Human breast adenocarcinoma (MDA-MB-231) and healthy fibroblast (L929) cell lines were purchased from the ATCC (American Type Culture Collection) and studied. Cells were mixed with 88% DMEM (Dulbecco’s Modified Eagle’s Medium; Gibco, Thermo Fisher Scientific), 10% FBS (Fetal Bovine Serum; Sigma-Aldrich), 1% glutamine (Sigma-Aldrich), and 1% penicillin (Sigma-Aldrich) solutions. The cells in which the medium was added were allowed to grow by incubating at 37 °C in an environment containing 95% humidity and 5% CO_2_. Before the MTT assay, the specimens were sterilized by Ethylene Oxide Sterilizer (Mallet Cats Engineering, Saverdun, France).

#### 2.5.2 Cell Viability Assay

The cytotoxic effects of all specimens, as determined by MTT analysis on L929 and MDA cell lines, were investigated. 6-well plates were used for seeding cells. Approximately 3 × 10^5^ cells were seeded in each well. Cells were allowed to adhere for 24 h, and then the specimens were cut into 2 × 2 cm pieces of the same dimensions and placed in each well plate. The wells were then incubated for 24 and 48 h. All wells without specimens were used as positive controls. After the incubation, the wells were treated with MTT solution to determine metabolically active cells and incubated at 37 °C for 3 h. After the MTT interaction, the wells were emptied, and DMSO solution was added to them. The formazan crystals formed were dissolved with this solution, and the number of viable cells in each well was determined by the color change. The absorbance values were read at 540 nm with the help of a microplate reader, and the values found were represented as mean ± standard deviation (±SD).

#### 2.5.3 Annexin V Binding Assay

Approximately 3 × 10^5^ seeds of each cell were seeded into 6-well plates and allowed to adhere overnight. The next day, sterilized specimens were placed in each well for 24 and 48 h. Cells harvested after trypsinization were suspended in PBS containing at least 1% FBS. The manufacturer’s instructions were then followed, and the Annexin V & Dead Cell reagent was mixed with the cells. Afterward, the percentages of dead, viable, early, and late apoptotic cells were determined using the Muse Cell Analyzer (Millipore) device.

## 3 Results and Discussion

### 3.1 Surface Morphology

The surface morphology and fiber diameter distribution of the electrospun mats are presented in Figure 1. All prepared mats—PCL/Cur, PCL/1nHAp-Cur, PCL/3nHAp-Cur, and PCL/5nHAp-Cur—exhibited smooth, bead-free, and randomly oriented fibers, confirming the stability of the electrospinning process. The incorporation of curcumin and n-HAp within the fibrous matrix did not cause any visible structural defects, such as bead formation or fiber fusion.

**Figure 1:**
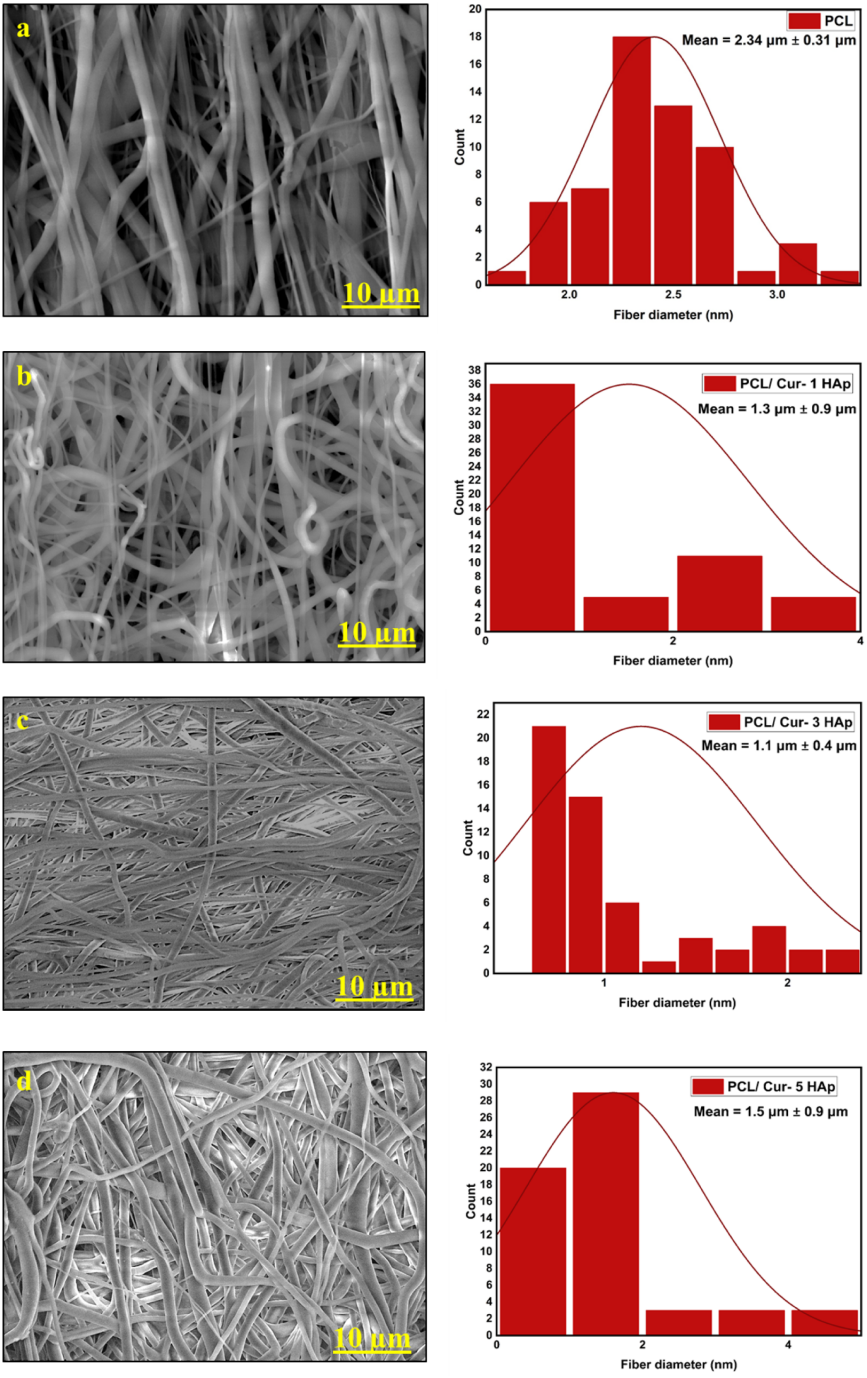
SEM micrographs and corresponding fiber diameter distributions of electrospun mats: (a) PCL/Cur, (b) PCL/1HAp-Cur, (c) PCL/3HAp-Cur, and (d) PCL/5HAp-Cur.

The average fiber diameters, calculated from histogram analyses of at least 50 measurements, were 2.34 ± 0.31 μm for PCL/Cur, 1.30 ± 0.90 μm for PCL/1HAp-Cur, 1.10 ± 0.40 μm for PCL/3HAp-Cur, and 1.50 ± 0.90 μm for PCL/5HAp-Cur. These values demonstrate a gradual decrease in fiber diameter with increasing n-HAp content up to 3 wt %, followed by a slight increase at 5 wt %. The observed reduction in fiber diameter can be attributed to the increased electrical conductivity of the spinning solution upon the addition of n-HAp, which enhances the elongation of the polymer jet under the applied electric field. At higher n-HAp loading (5 wt %), the slight diameter increase may result from nanoparticle agglomeration, leading to higher solution viscosity and reduced jet stretching.

The SEM observations demonstrated that all electrospun mats possessed continuous, bead-free, and well-defined fibrous structures, indicating the successful optimization of the electrospinning parameters. The gradual reduction in fiber diameter with increasing n-HAp concentration up to 3 wt % suggests that the incorporation of conductive nanoparticles improved the jet stretching behavior during electrospinning. This enhancement can be attributed to the increased charge density of the spinning solution, which facilitates finer fiber formation. However, the slight increase in diameter observed at 5 wt % n-HAp may arise from nanoparticle agglomeration, leading to higher viscosity and limited jet elongation. Previous studies have reported similar behavior, where increasing polymer content or nanoparticle aggregation affected fiber uniformity and diameter distribution.^25,26^ The results indicate that an optimal n-HAp concentration (3 wt %) provides improved solution stability and uniform electrospinning, yielding finer fibers with a narrow diameter distribution. Overall, the SEM analysis confirmed that both curcumin and n-HAp were successfully incorporated into the PCL matrix, forming defect-free, randomly oriented microfibers with a continuous morphology suitable for biomedical drug delivery applications.

### 3.2 FTIR Analysis

The FTIR spectra of n-HAp, Cur, and the electrospun mats (PCL/Cur, PCL/1HAp-Cur, PCL/3HAp-Cur, and PCL/5HAp-Cur) are shown in Figure 2.

**Figure 2:**
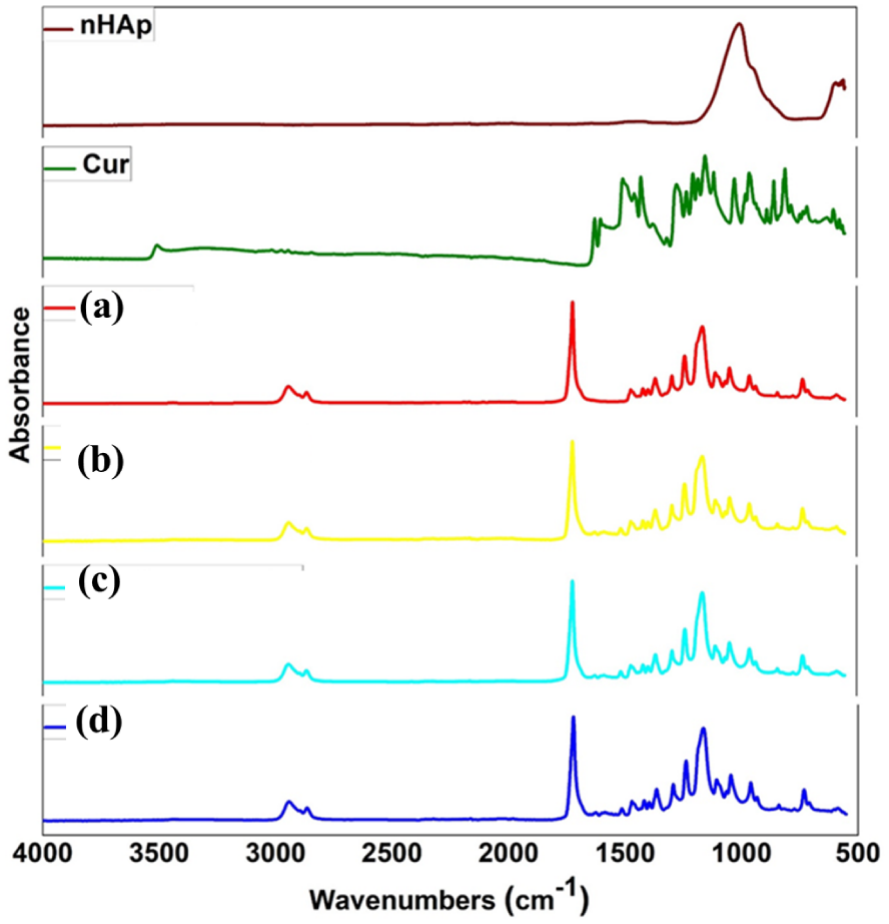
FTIR spectra of n-HAp, curcumin, and electrospun mats: (a) PCL/Cur, (b) PCL/1HAp-Cur, (c) PCL/3HAp-Cur, and (d) PCL/5HAp-Cur.

All characteristic peaks corresponding to the polymer matrix, curcumin, and n-HAp were clearly identified, confirming the successful incorporation of the components without chemical degradation during electrospinning. For PCL/Cur, the characteristic absorption bands of PCL were observed at 2943 cm^–1^ and 2866 cm^–1^ (asymmetric and symmetric CH_2_ stretching), 1720 cm^–1^ (C=O stretching of the ester group), and 1240–1160 cm^–1^ (C–O–C stretching vibrations).^27^ These peaks indicate that the semicrystalline nature of PCL was preserved after electrospinning.

The FTIR spectrum of Curcumin displayed distinct bands at 1627 cm^–1^ (C=O stretching of the enolic group and C=C stretching in the aromatic ring), 1598 cm^–1^ (aromatic C=C stretching), 1508–1460 cm^–1^ (olefinic C–H bending), and 1275–1020 cm^–1^ (C–O and C–O–C stretching).^28^ These peaks were clearly visible in all curcumin-loaded mats, confirming that curcumin was successfully incorporated into its molecular form without structural alteration. For n-HAp, characteristic phosphate (PO_4_^3^ −) vibrations were observed at 1090 cm^–1^ (ν_3_), 962 cm^–1^ (ν_1_), and 603–563 cm^–1^ (ν_4_), corresponding to the stretching and bending modes of phosphate groups.

In the composite mats, these peaks overlapped partially with PCL’s C–O–C vibrations but were still detectable, indicating the homogeneous dispersion of n-HAp in the PCL matrix. The weak intensity of these phosphate bands is consistent with the relatively low filler loading (1–5 wt %), as also reported in similar polymer–hydroxyapatite systems.^26,29^

Importantly, no new absorption peaks or major band shifts were observed in any of the composites, suggesting that the interaction among curcumin, n-HAp, and PCL was primarily physical rather than chemical.

The spectra confirm that curcumin and n-HAp were well embedded within the polymer network without altering the intrinsic chemical structure of PCL, indicating that the electrospinning process maintained the molecular stability of all components.^23,30^

### 3.3 XRD Analysis

The crystalline structure of the electrospun mats was analyzed by X-ray diffraction (XRD), and the corresponding diffractograms are presented in Figure 3. The characteristic diffraction peaks of the PCL appeared at 2θ = 21.3° and 23.6°, corresponding to the (110) and (200) crystal planes of its orthorhombic lattice, which confirms the semicrystalline nature of the polymer.^23,31^ These reflections were clearly visible in all samples, indicating that the electrospinning process preserved the crystalline domains of the PCL.

**Figure 3:**
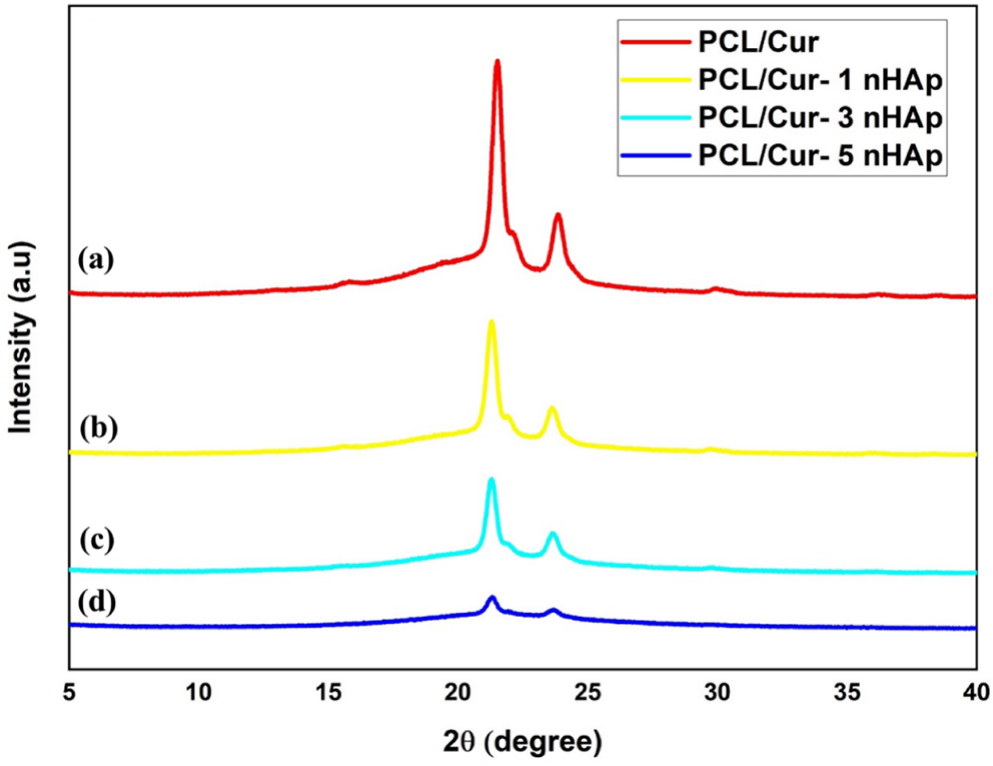
XRD diffractograms of electrospun mats: (a) PCL/Cur, (b) PCL/1HAp-Cur, (c) PCL/3HAp-Cur, and (d) PCL/5HAp-Cur.

With the incorporation of n-HAp nanoparticles (1–5 wt %), the overall diffraction pattern retained the same characteristic peaks of PCL, with only a slight decrease in peak intensity and broadening at higher n-HAp content. This reduction in intensity can be attributed to the disruption of long-range crystalline ordering in PCL chains due to the uniform dispersion of n-HAp within the polymer matrix. The typical diffraction peaks of crystalline hydroxyapatite, generally observed around 2θ = 25.9°, 31.7°, and 34.0° (corresponding to the (002), (211), and (202) planes), were not distinctly visible in the composites. This absence indicates that n-HAp was well distributed in the amorphous regions of PCL and did not form large crystalline aggregates.^26,32^

Moreover, the diffraction pattern of PCL/Cur exhibited no additional crystalline peaks from curcumin, suggesting that curcumin was present in an amorphous or molecularly dispersed state within the PCL matrix. The suppression of curcumin’s characteristic crystalline reflections between 10° and 30° implies improved solubility and homogeneous distribution of the drug in the fibrous structure.^30,33^

In summary, XRD analysis confirmed that the electrospun mats maintained the semicrystalline nature of PCL, while both curcumin and n-HAp were incorporated in an amorphous or highly dispersed form. The slight reduction in crystallinity with increasing n-HAp concentration is consistent with the morphological and FTIR results, suggesting good compatibility and uniform blending among the polymer, nanoparticle, and drug phases.

### 3.4 Contact Angle Analysis

The surface wettability of the electrospun mats was evaluated by using static water contact angle (CA) measurements, and the results are shown in Figure 4. The contact angle values of the fabricated samples were 132 ± 0.8° (PCL/Cur), 123 ± 2.3° (PCL/1nHAp-Cur), 112 ± 1.8° (PCL/3nHAp-Cur), and 128 ± 0.7° (PCL/5nHAp-Cur).

**Figure 4:**
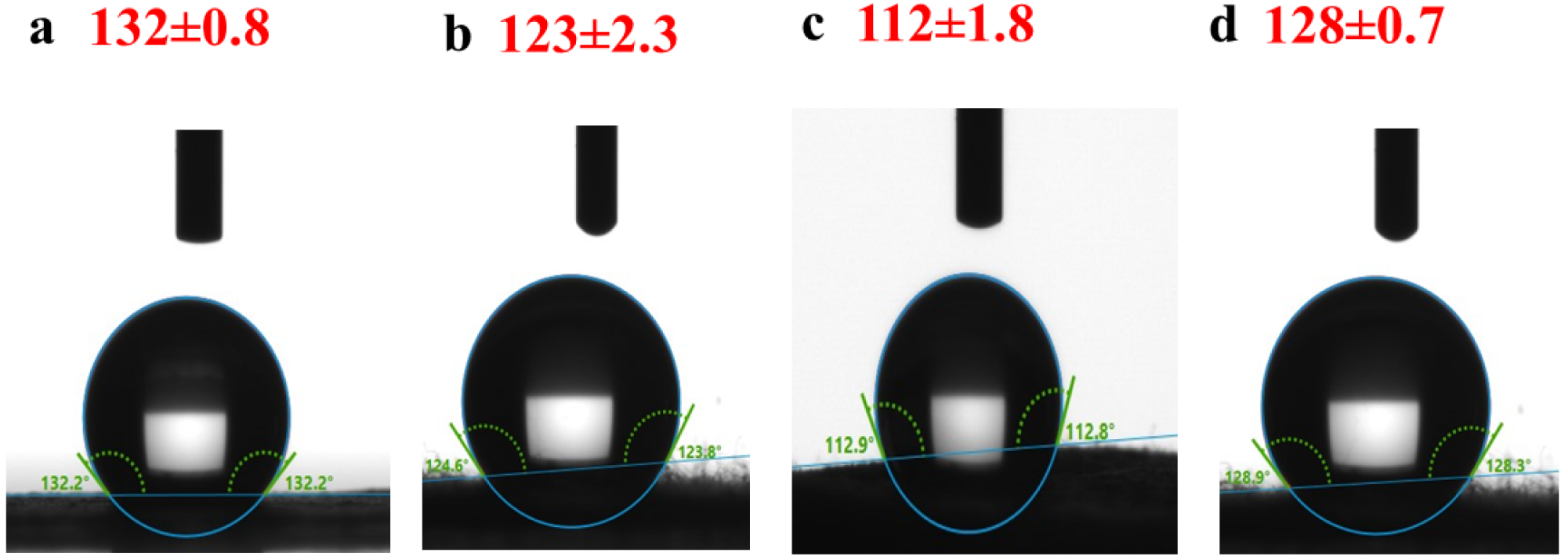
Contact angle images and values of electrospun mats: (a) PCL/Cur, (b) PCL/1HAp-Cur, (c) PCL/3HAp-Cur, and (d) PCL/5HAp-Cur.

All samples exhibited contact angles above 90°, confirming their predominantly hydrophobic character, which is typical for PCL-based materials.^34^ However, the incorporation of n-HAp significantly influenced the surface wettability. The addition of 1 wt % n-HAp slightly decreased the contact angle compared to pure PCL/Cur, indicating a mild enhancement in surface hydrophilicity due to the presence of hydrophilic phosphate and hydroxyl groups on n-HAp particles. The lowest contact angle was recorded for PCL/3HAp-Cur (112 ± 1.8°), suggesting that this composition provided the most balanced distribution of n-HAp at the fiber surface, promoting greater water affinity.

At higher loading (5 wt % n-HAp), the contact angle increased again to 128 ± 0.7°, likely due to ceramic phase agglomeration within the fibers, reducing exposed hydrophilic sites. Similar nonlinear behavior has been reported for hydroxyapatite-modified electrospun composites, where excessive particle content decreases effective surface energy by promoting surface roughness and hydrophobic domain formation.^26^

Overall, the results demonstrate that the controlled addition of n-HAp can modulate the surface wettability of PCL-based electrospun mats. The moderately reduced contact angle observed for PCL/3HAp-Cur indicates improved hydrophilicity and a potentially enhanced cell attachment capability, which are advantageous for biomedical applications such as tissue regeneration and local drug delivery.

Although all electrospun mats exhibited water contact angles above 90°, cell attachment was still observed, particularly for the PCL/3HAp-Cur sample. This behavior may be associated with the fibrous topography of electrospun mats, which provides a high surface area and micro/nanoscale roughness that can support focal adhesion formation even on moderately hydrophobic surfaces.^35^ In addition, the incorporation of nanohydroxyapatite introduces phosphate and hydroxyl groups that may facilitate serum protein adsorption, thereby promoting integrin-mediated cell–material interactions.^36^ Furthermore, bioactive PCL-based composite electrospun fibers have demonstrated favorable cell responses despite similar contact angle values in recent studies, suggesting that wettability alone does not fully determine cell adhesion.^37^

### 3.5 Drug Release Behavior and Kinetic Evaluation

The in vitro release profile of curcumin (Cur) from the electrospun mats was investigated in PBS (pH 7.4) containing 0.5 wt % Tween 80 at 37 °C, and the results are shown in Figure 5.

**Figure 5:**
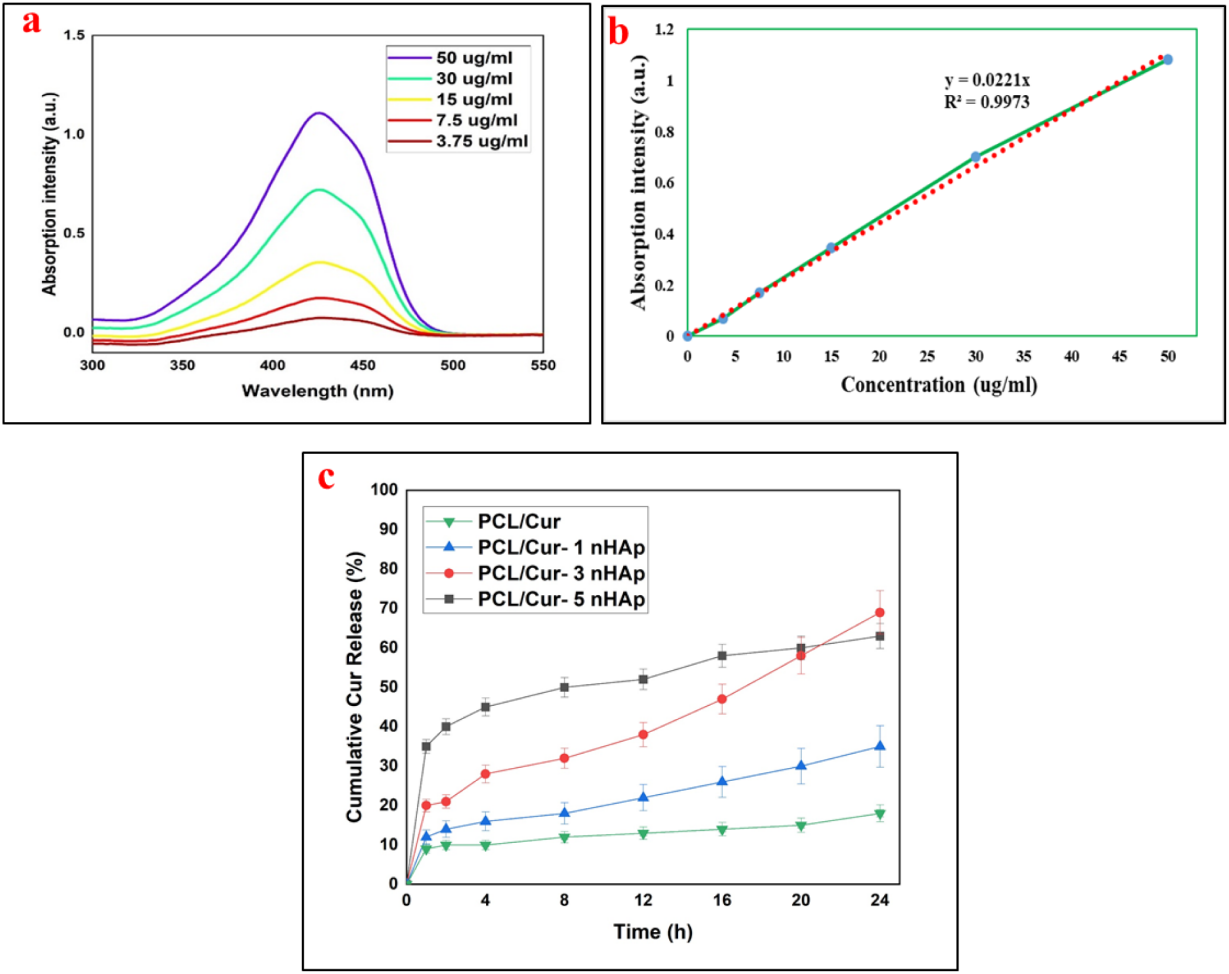
(a) UV–Vis spectra of Cur calibration solutions (3.75–50 μg mL^–1^); (b) calibration curve with linear fit (*R* ^2^ = 0.9973); (c) Cumulative Cur release from electrospun mats: (black square) PCL/Cur, (blue traingle) PCL/1HAp-Cur, (red circle) PCL/3HAp-Cur, (green inverted triangle) PCL/5HAp-Cur at 37 °C in PBS (pH 7.4).

The UV–Vis spectra of Cur solutions with different concentrations (3.75–50 μg mL^–1^) exhibited a characteristic absorption maximum at ≈425 nm (Figure 5a). A calibration curve constructed at this wavelength (Figure 5b) showed an excellent linear relationship between absorbance and concentration (*y* = 0.0221x, *R* ^2^ = 0.9973), confirming the reliability of quantitative analyses.

The cumulative Cur release from the mats (Figure 5c) followed a biphasic pattern characterized by an initial burst, followed by a sustained release phase. After 24 h, the cumulative release reached approximately 20% (PCL/Cur), 30% (PCL/1HAp-Cur), 60% (PCL/3HAp-Cur), and 80% (PCL/5HAp-Cur). The presence of n-HAp markedly enhanced Cur diffusion from the PCL matrix, particularly at higher loadings.

This effect is attributed to the increased surface hydrophilicity and interfacial porosity induced by n-HAp, which facilitate the penetration of the aqueous medium and accelerate drug diffusion through the polymer network. The moderate release rate observed for PCL/3HAp-Cur indicates an optimal balance between polymer–drug interaction and diffusion control, whereas the faster release from PCL/5HAp-Cur may result from local nanoparticle agglomeration that generates microdefects and increases pathway connectivity. Such nonlinear dependence of release rate on n-HAp loading within the fibrous matrix has been reported previously for PCL-based composites, where excessive filler concentrations reduce matrix uniformity.^10,23,38^ The results suggest that the addition of moderate amounts of n-HAp can effectively tailor the release kinetics of curcumin, providing a sustained-release platform with the potential for prolonged local therapeutic action.

To elucidate the release mechanism, the data were fitted to several kinetic models—Weibull, Makoid–Banakar, Peppas–Sahlin, and Korsmeyer–Peppas—and the obtained parameters are summarized in Table 2. Among them, the Weibull model provided the best fit for all systems, with *R* ^2^ = 0.94–0.99, indicating a nonlinear diffusion-controlled process.

**Table 2:** Release Kinetic Parameters for Curcumin-Loaded Electrospun Mats Based on Different Kinetic Models

		Electrospun delivery systems			
Model	Parameters	PCL/Cur	PCL/1HAp-Cur	PCL/3HAp-Cur	PCL/5HAp-Cur
Weibull	*R* ^2^	0.982	**0.946**	**0.997**	0.948
	α	7.852	**2.659**	**1.893**	4.556
	β	0.245	**0.184**	**0.039**	0.209
	*T*_i_	0.479	**0.490**	**0.498**	0.000
Makoid–Banakar	*R* ^2^	0.981	0.921	0.986	**0.985**
	*K*_MB_	9.176	25.519	38.627	**21.130**
	*n*	0.387	0.277	0.055	**0.078**
	*k*	0.007	0.007	0.001	**–0.006**
Peppas–Sahlin	*R* ^2^	**0.983**	0.929	0.905	0.905
	*k*_1_	**9.982**	29.391	14.292	41.262
	*k*_2_	**–0.865**	–3.993	5.438	–7.294
	*m*	**0.436**	0.343	0.133	0.342
Korsmeyer-Peppas	*R* ^2^	0.952	0.889	0.986	0.954
	*K*_KP_	10.302	27.212	38.753	19.724
	*n*	0.259	0.176	0.047	0.176

For PCL/3HAp-Cur, the highest *R* ^2^ (≈0.997) and lowest α (≈ 1.9) values suggest a faster initial release followed by sustained diffusion, consistent with the moderate wettability of this composition. The β values below 0.25 indicate that Cur release is primarily governed by Fickian diffusion. The Korsmeyer–Peppas model further confirmed this behavior with diffusional exponents (*n*) < 0.45 for all mats, signifying diffusion-controlled transport through the polymeric matrix rather than erosion- or swelling-driven release.

Overall, the combined results demonstrate that Cur release from the electrospun PCL/n-HAp composites follows a biphasic, diffusion-dominated mechanism, where the addition of n-HAp effectively modulates both the rate and extent of drug liberation. This tunable release behavior, together with the structural stability of the mats, highlights the potential of PCL/3HAp-Cur as an optimized formulation for sustained local therapeutic delivery.

The three-phase release profile of PCL/3HAp-Cur can be attributed to the distribution of curcumin and the structural characteristics of the composite. The initial rapid release is associated with surface-located curcumin, whereas the subsequent slower phase reflects diffusion through semicrystalline PCL regions, where restricted chain mobility limits transport.^7^ The later reacceleration is related to the hydrophilicity of nanohydroxyapatite, which promotes progressive water uptake and microporosity formation, resulting in localized polymer relaxation and the opening of secondary diffusion pathways.^39^ Such multistep profiles are consistent with diffusion–relaxation mechanisms described for polymeric drug delivery systems.^40^

### 3.6 In Vitro Cell Viability

To evaluate the cell viability of the fabricated fibrous mats on the breast cancer cell line (MDA), an MTT assay was performed, and the results are illustrated in Figure 5. The blank PCL displayed no cytotoxicity on MDA cells, indicating that PCL was biocompatible and the feasibility of its application as drug carriers. The results demonstrated that the viabilities of MDA cells obtained after 24 h incubation were 82.06 ± 1.53%, 68.66 ± 2.68%, and 85.89 ± 1.34% for the electrospun mats of PCL-Cur-1 nHAp, PCL-Cur-3 nHAp, and PCL-Cur-5 nHAp, respectively, while 65.08 ± 2.65%, 50.55 ± 2.17%, and 70.40 ± 1.88% of cells remained viable after 48 h incubation. On the other hand, the viabilities of L929 cells obtained after 24 h incubation were 99.35 ± 0.53%, 97.42 ± 2.08%, and 101.94 ± 2.34% for the electrospun mats of PCL-Cur-1 nHAp, PCL-Cur-3 nHAp, and PCL-Cur-5 nHAp, respectively, while 89.81 ± 1.65%, 78.42 ± 3.17%, and 87.33 ± 2.88% of cells remained viable after 48 h incubation.

Morphological changes in MDA-MB-231 and L929 cells after treatment with electrospun mats were examined under an inverted optical microscope, and the representative images are presented in Figure 7. Panels (a–d) correspond to the same sample order as in Figure 6. In the untreated control group, MDA-MB-231 cells displayed a dense, spindle-shaped morphology with uniform adherence. In contrast, after exposure to the curcumin-loaded mats, marked changes were observed—including cell shrinkage, membrane blebbing, and loss of confluence—especially in (c) PCL/3HAp-Cur, which exhibited the most severe apoptotic morphology. These structural changes indicate apoptosis induction rather than necrotic death, consistent with curcumin’s known pro-apoptotic pathways involving caspase activation and mitochondrial depolarization.

**Figure 6:**
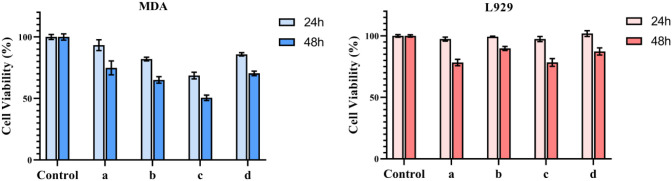
Cell viability of MDA-MB-231 breast cancer cells and L929 fibroblast cells after 24 and 48 h of incubation with electrospun mats: (a) PCL/Cur, (b) PCL/1HAp-Cur, (c) PCL/3HAp-Cur, and (d) PCL/5HAp-Cur.

L929 fibroblast cells, however, retained their normal polygonal morphology, with intact membranes in all treated groups, confirming the biocompatibility of the PCL/n-HAp/Cur composite systems. In addition, it should be noted that, in our system, nanohydroxyapatite is presented in an incorporated PCL micro/nanofibrous matrix rather than as a free nanoparticle suspension; therefore, its cytotoxic behavior is more appropriately interpreted in the context of polymer–ceramic composite scaffolds, which have been widely reported to support high cell viability in vitro,^41–44^ while studies on isolated n-HAp suspensions indicate a size- and dose-dependent response that is not directly comparable to our configuration.^45,46^ This selective apoptotic behavior aligns with previous studies demonstrating the synergistic cytotoxic and osteoconductive effects of curcumin and nanohydroxyapatite Figure 7.^23,47^

**Figure 7:**
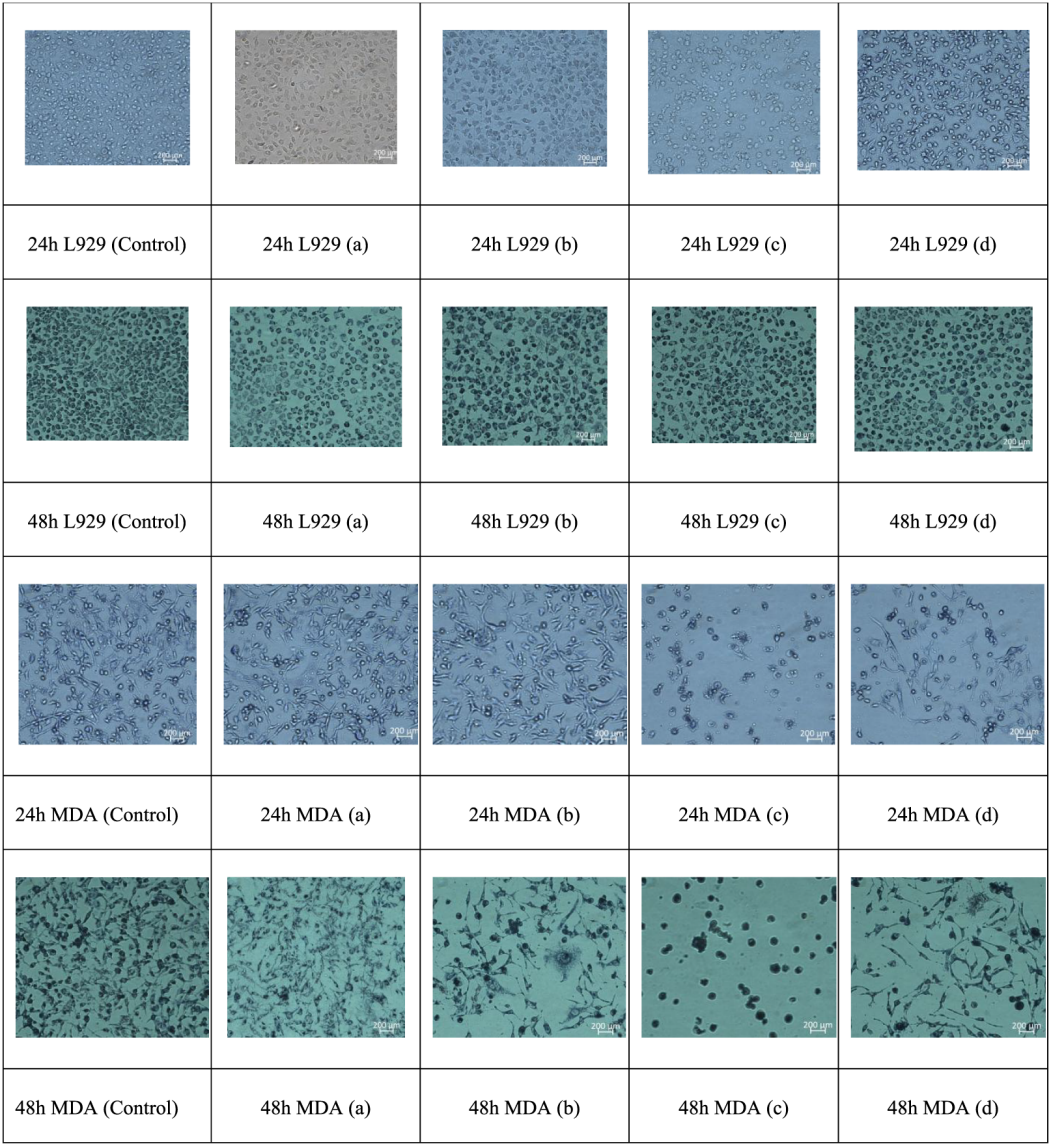
Representative optical micrographs of MDA and L929 cells exposed to (a) PCL/Cur, (b) PCL/1HAp-Cur, (c) PCL/3HAp-Cur, and (d) PCL/5HAp-Cur for 24 and 48 h.

### 3.7 Annexin V Binding Assay

Following the MTT results, PCL/3HAp-Cur was selected as the most effective formulation for apoptotic analysis. The induction of apoptosis in MDA-MB-231 breast cancer cells was evaluated by using the Annexin V-FITC/PI flow cytometry method (Figure 8).

**Figure 8:**
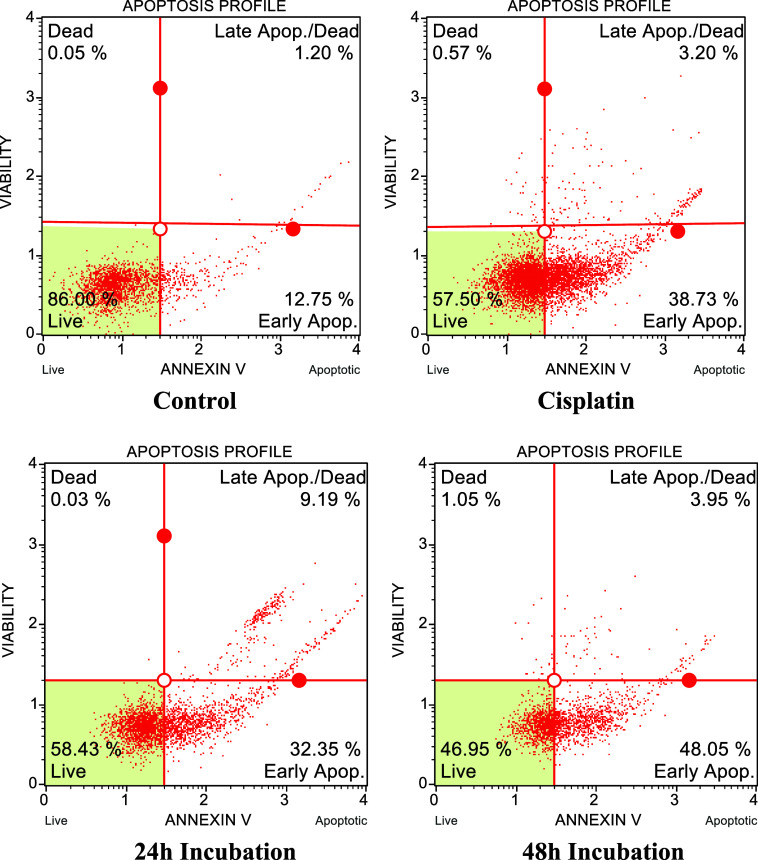
Flow cytometry results for MDA cell line with the specimen of PCL-Cur-3 nHAp (24–48 h incubation), control, and cisplatin.

Four experimental groups were compared: (a) Control, (b) Cisplatin-treated positive control, (c) 24 h of incubation with PCL/3HAp-Cur, and (d) 48 h of incubation with PCL/3HAp-Cur.

The flow cytometric histograms revealed a distinct shift from viable to apoptotic cell populations in the treated samples. After 24 h of exposure to PCL/3HAp-Cur (c), the proportion of early apoptotic cells increased markedly compared to the control group. After 48 h (d), a significant rise in both early and late apoptosis fractions was detected, accompanied by a notable reduction in viable cells, confirming a strong time-dependent pro-apoptotic effect.

When compared with cisplatin (b)—a clinically established chemotherapeutic drug for breast cancer—the 48 h PCL/3HAp-Cur treatment demonstrated a higher percentage of early apoptotic cells, indicating a comparable or even superior apoptotic efficacy, potentially reducing the systemic toxicity commonly associated with conventional chemotherapy, as expected for localized drug delivery approaches.^48^

These findings suggest that the enhanced apoptosis is primarily driven by sustained curcumin release and possible Ca^2+^ /ROS-related pathways associated with nanohydroxyapatite, as suggested in previous studies,^46^ which synergistically trigger mitochondrial-dependent apoptosis pathways.

Overall, the flow cytometry results corroborate the MTT assay outcomes, confirming that the hybrid PCL/3HAp-Cur mat induces selective apoptotic cell death in MDA-MB-231 cells while maintaining high viability and normal morphology of healthy fibroblasts under the tested conditions, consistent with the standard in vitro cytotoxicity criteria (ISO 10993-5).

The obtained data showed that the MDA cell line undergoes early apoptosis as a result of 24 and 48 h incubations of PCL-Cur-3 nHAp. Cisplatin is an effective treatment drug currently used in the treatment of breast cancer.^49,50^ When the results obtained with this drug were compared, it was determined that the rate of early apoptosis after 48 h of incubation was higher than cisplatin.

## 4 Conclusion

In this study, electrospun PCL/Cur, PCL/1 nHAp-Cur, PCL/3 nHAp-Cur, and PCL/5 nHAp-Cur composite fibrous mats were successfully fabricated by the electrospinning technique for potential use in drug delivery applications. SEM analysis confirmed the formation of uniform, randomly oriented, and bead-free fibrous structures for all of the fabricated mats. The average fiber diameters were found to decrease with the incorporation of n-HAp up to 3 wt %, followed by a slight increase at 5 wt %, indicating the effect of n-HAp loading within the fibers on fiber morphology. FTIR and XRD characterizations demonstrated that the characteristic peaks of PCL, n-HAp, and curcumin were retained without new chemical bond formation, confirming that both additives were successfully incorporated into the polymer matrix and were well dispersed at the nanoscale. Contact angle measurements revealed improved hydrophilicity for the PCL/3HAp-Cur sample, which also exhibited the most homogeneous fiber structure.

The in vitro drug release results showed a biphasic release profile, with cumulative curcumin release after 24 h increasing from 20 to 80% as the n-HAp content increased from 0 to 5 wt %. The PCL/3HAp-Cur composition exhibited a more sustained release pattern compared to that of other groups, suggesting an optimal balance between diffusion and matrix interaction. Kinetic modeling confirmed a diffusion-controlled mechanism, best fitting the Weibull equation. MTT assay results indicated that all curcumin-loaded mats exhibited time-dependent cytotoxicity against MDA-MB-231 breast cancer cells, with the PCL/3HAp-Cur group showing the highest anticancer activity while maintaining high viability toward L929 fibroblast cells. Moreover, Annexin V/PI flow cytometry analysis verified that apoptosis was the main mechanism induced by PCL/3HAp-Cur.

In conclusion, the relationship between curcumin release and n-HAp incorporation did not follow a linear trend; instead, an intermediate level of n-HAp (3 wt %) provided the most favorable performance in terms of sustained drug release, improved hydrophilicity, and enhanced anticancer efficacy. Therefore, adjusting the n-HAp content within the polymer matrix offers an effective approach to controlling the drug release profile and increasing the therapeutic potential of curcumin-loaded electrospun mats for localized cancer treatment.

From an application standpoint, the curcumin-loaded PCL/n-HAp hybrid microfibers may be suitable for localized breast cancer therapy, particularly in postsurgical tumor resection cavities requiring sustained drug release. The implantable microfiber structure may help reduce systemic exposure compared to conventional chemotherapy. The presence of n-HAp may further support tissue interaction in tumor-adjacent bone involvement scenarios. Additional in vivo studies are needed to confirm its translational applicability.
